# The Evolutionary Conserved SWI/SNF Subunits ARID1A and ARID1B Are Key Modulators of Pluripotency and Cell-Fate Determination

**DOI:** 10.3389/fcell.2021.643361

**Published:** 2021-03-04

**Authors:** Luca Pagliaroli, Marco Trizzino

**Affiliations:** Department of Biochemistry and Molecular Biology, Sidney Kimmel Medical College, Thomas Jefferson University, Philadelphia, PA, United States

**Keywords:** chromatin remodeling, SWI/SNF, ARID1A, ARID1B, development, pluripotency, cell identity

## Abstract

Organismal development is a process that requires a fine-tuned control of cell fate and identity, through timely regulation of lineage-specific genes. These processes are mediated by the concerted action of transcription factors and protein complexes that orchestrate the interaction between *cis*-regulatory elements (enhancers, promoters) and RNA Polymerase II to elicit transcription. A proper understanding of these dynamics is essential to elucidate the mechanisms underlying developmental diseases. Many developmental disorders, such as Coffin-Siris Syndrome, characterized by growth impairment and intellectual disability are associated with mutations in subunits of the SWI/SNF chromatin remodeler complex, which is an essential regulator of transcription. *ARID1B* and its paralog *ARID1A* encode for the two largest, mutually exclusive, subunits of the complex. Mutations in *ARID1A* and, especially, *ARID1B* are recurrently associated with a very wide array of developmental disorders, suggesting that these two SWI/SNF subunits play an important role in cell fate decision. In this mini-review we therefore discuss the available scientific literature linking *ARID1A* and *ARID1B* to cell fate determination, pluripotency maintenance, and organismal development.

## Introduction

### The SWI/SNF Complex

**FIGURE 1:**
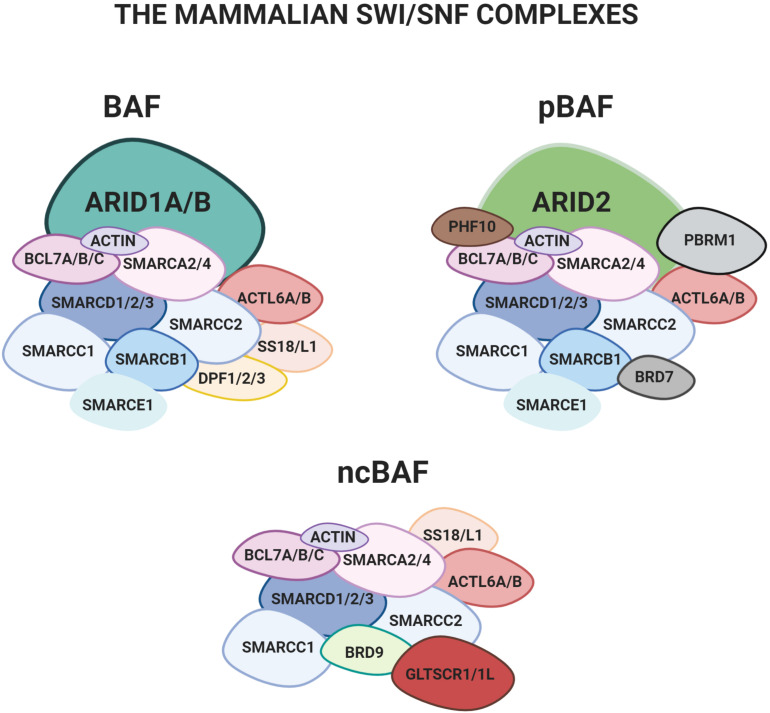
The three main configurations of the mSWI/SNF complex: BAF, pBAF e ncBAF. The two mutually exclusive subunits ARID1A and ARID1B, object of the present review, are only found in the BAF.

The SWI/SNF (SWItch/Sucrose Non-Fermentable) chromatin remodeling complex leverages an ATP-dependent mechanism to modify the structure of the chromatin and modulate its accessibility to transcriptional regulators (Figure 1). It was first discovered in yeast (SWI/SNF) (Stern et al., 1984), later in *Drosophila* (Brm-associated protein, BAP) (Kennison and Tamkun, 1988; Tamkun et al., 1992) and finally in mammals (Brg/Brahma-associated factors, BAF) (Wang et al., 1996).

In mammals, the different subunits comprise eight different bromodomains, two PHD finger proteins, two chromodomain and multiple proteins with DNA binding domains (Wang et al., 1996a,b; Wang et al., 1998; Lessard et al., 2007). These various subunits are not always present at the same time.

Mammalian SWI/SNF (mSWI/SNF) complexes are assembled from subunits encoded by 29 genes, including multiple paralogs, which generate an extensive diversity in composition. Three versions of the mSWI/SNF were recently characterized in detail: 1) BRG1/BRM-associated factor complex (BAF), 2) polybromo containing complex (pBAF), and 3) a non-canonical version of the complex (ncBAF) (Figure 1) (Mashtalir et al., 2018).

The SWI/SNF complex is able to modify the structure of the chromatin leveraging the energy generated by the hydrolysis of ATP. SWI/SNF binds to the nucleosome in a central cavity where the DNA is exposed (Havas et al., 2000; Saha et al., 2005). Once bound, the complex uses the energy derived by the ATP hydrolysis to break the binding between histones and DNA, promoting the formation of a transient DNA loop that spreads around the nucleosome, ultimately orchestrating the changes in chromatin accessibility (Dechassa et al., 2008; Tang et al., 2010; Tyagi et al., 2016). As a consequence of this process, the chromatin becomes more accessible and permissive to the binding of transcription factors (Kadoch et al., 2017).

All the existing mammalian configurations of the complex contain an ATPase subunit, either SMARCA4 (BRG1) or SMARCA2 (BRM), which catalyzes the hydrolysis of ATP. Several other “core-subunits” are shared by all the different configurations (e.g., SMARCD1/2/3, SMARCC1/2 and a few others; Mashtalir et al., 2018). Finally, some of the subunits are only present in specific configurations. Among these, the mutually exclusive AT-rich interactive domain proteins ARID1A or ARID1B are only found in the BAF (Wilsker et al., 2004; Patsialou et al., 2005; Raab et al., 2015; Mashtalir et al., 2018).

ARID1A and ARID1B are conserved throughout metazoans and expressed across most human cells and tissues. Mutations in the genes encoding for these two subunits are associated with a wide array of developmental disorders and cancers, suggesting that they are implicated in the maintenance of cell identity and in the determination of cell fate. Based on this premise, in the present review, we discuss the role of ARID1A and ARID1B in the maintenance of pluripotency, in the determination of cell fate, and, more broadly, in organismal development.

### Mutations in *ARID1A*

*ARID1A* encodes for the AT-Rich Interactive Domain-containing protein 1A (ARID1A/BAF250a). It is the most frequently mutated member of the SWI/SNF family in cancer. *ARID1A* mutations are associated with a wide range of cancers, including ovarian endometrioid/clear-cell carcinomas, pancreatic cancer, gastric carcinoma, esophageal adenocarcinoma, renal carcinoma and breast tumors (Biegel et al., 2014; Wu et al., 2014; Kadoch and Crabtree, 2015; Masliah-Planchon et al., 2015; Takeda et al., 2016). Despite this evidence, the role of ARID1A in cancer is still not fully understood, with some studies suggesting a tumor suppression role, while a few others indicate an oncogenic function (Fang et al., 2015; Gibson et al., 2016; Zhai et al., 2016; Zhao et al., 2016; Mathur et al., 2017). In the case of endometrial and ovarian cancers, these mutations might either hamper the nuclear import of ARID1A, or affect the ability of ARID1A to interact with the subunits of SWI/SNF complex (Guan et al., 2012). The most frequently dysregulated pathway is PI3K/AKT, along with the downstream signaling cascades PTEN and PIKC3A (Takeda et al., 2016).

Mutations in *ARID1A* also lead to Coffin-Siris Syndrome, a neurodevelopmental disorder which will be further discussed in this review (Santen et al., 2013; Wieczorek et al., 2013; Kosho et al., 2014; Tsurusaki et al., 2014; Lee and Ki, 2021). *ARID1A* mutations are typically frame-shift or nonsense, spread across the gene with no specific hotspots, resulting in loss of protein level.

### Mutations in *ARID1B*

*ARID1B* encodes for the AT-rich interactive domain-containing protein 1B (ARID1B/BAF250b). *ARID1B* mutations are commonly associated with neurodevelopmental disorders. Most frequently, *ARID1B* mutations are *de novo* haploinsufficient mutations, with no specific gene hotspots.

To date, the majority of the reported mutations are either nonsense or frameshift. These mutations result in non-functional truncated proteins, triggering *ARID1B*-haploinsufficiency and associated pathologies (Schweingruber et al., 2013; Sim et al., 2015). Mutations in *ARID1B* may disrupt the ability of the SWI/SNF complex to bind the chromatin (Sim et al., 2015).

Often, *ARID1B* mutations result in Coffin-Siris Syndrome, a relatively rare genetic disorder that manifests at birth and is characterized by both intellectual disabilities and physical phenotypes (Coffin and Siris, 1970; van der Sluijs et al., 2019). Coffin-Siris patients usually show coarse facial features, impaired craniofacial development, and hypoplastic fifth finger nails (Schrier et al., 2012). Further, most individuals present mild to severe intellectual disability, speech impairment and impaired motor skills (Vergano and Deardorff, 2014). Other characteristics of this disorder include respiratory infections, feeding issues, hearing loss, sparse scalp hair and hypermobility of joints (Vergano and Deardorff, 2014). Mutations in *ARID1B* have also been linked to Autism Spectrum Disorder (ASD), Intellectual Disabilities (ID), epilepsy and neuroblastoma (Vergano et al., 1993; Halgren et al., 2012; Hoyer et al., 2012; Santen et al., 2012; Vals et al., 2014; Yu et al., 2015; Ben-Salem et al., 2016; Sonmez et al., 2016; Jung et al., 2017; Lee et al., 2017; Shibutani et al., 2017; Yu et al., 2018; Demily et al., 2019; Filatova et al., 2019; Pranckeniene et al., 2019; Sekiguchi et al., 2019; van der Sluijs et al., 2019; Curcio et al., 2020; Fujita et al., 2020; Lian et al., 2020; Pascolini et al., 2020; Smith et al., 2020). *ARID1B* mutations can be associated with both syndromic and non-syndromic forms of ID (van der Sluijs et al., 2019). In this context, Coffin-Siris patients almost always show some degree of ID, and often present some characteristics that can be associated to Autism Spectrum Disorder. Recently, van der Sluijs et al. (2019) sought to determine genotypic and phenotypic differences between ARID1B-ID and ARID1B-CSS. They found only minor differences between ARID1B-ID and ARID1B-CSS patients, and suggested that ARID1B-related disorders seem to consist of a spectrum, and patients should be managed similarly.

Several studies tried to uncover genes and pathways most commonly dysregulated in ARID1B-ID and ARID1B-CSS. A recent paper looked at gene expression in monocytes of CSS patients. The study identified few differentially expressed genes (*CRYZ*, *TRGV5*, *TSPAN33*, *TPPP3*, *SAMD9L*, *DDX60*, *FMN1*, *PER1*, *MIR3648*, and *GSTM1*) (Kalmbach et al., 2019) and the pathway analysis did not reveal any statistically significant network. A previous study investigated gene expression in a single CSS patient carrying a novel microduplication of *ARID1B*, and identified EIF2 signaling and the regulation of eIF4 and p70S6K signaling as top canonical pathways (Seabra et al., 2017). Using an *ARID1B*-haploinsufficient mouse model, Celen et al. (2017) detected dysregulations in the Ephrin, nNOS, axonal guidance and glutamate receptor signaling pathways. Gene expression profile performed by Shibutani et al. (2017) suggested that *ARID1B^+/–^* mice exhibit a pattern very similar to autistic brains centered on immature fast spiking cells. Amongst the several differentially expressed genes, *HOXB2*, *PRL*, *PODNL1*, and *PTH2* were the most downregulated, whereas *AREG*, *GBP8*, *KLR2*, and *ZP2* were the most upregulated (Shibutani et al., 2017).

### The Role of ARID1A and ARID1B in Pluripotency and Cell Fate Determination

The contribution of ARID1A and ARID1B to cell pluripotency has been predominantly investigated in mouse embryos and in embryonic stem cells (ESCs). These cells are distinguished by their ability to differentiate into almost any cell lineage.

Gao et al. (2008) demonstrated that embryos carrying a homozygous *ARID1A* knockout are able to differentiate in primitive endoderm and epiblast layers but are unable to generate the mesodermal layer. Moreover, *ARID1A*^–/–^ mouse ESCs fail to maintain a normal stem cell phenotype in culture and spontaneously differentiate (Gao et al., 2008). These pluripotency anomalies seem to be lineage specific, since the ESCs cannot differentiate into cardiomyocytes or adipocytes, but can differentiate into ectoderm-derived neurons (Gao et al., 2008). Consistent with this, Lei et al. (2015) observed dysregulated expression of key developmental and pluripotency genes in *ARID1A*^–/–^ mouse ESCs. In particular, the most frequently affected genes were associated with the generation of the mesodermal and endodermal layers (Lei et al., 2015).

Similar results were published by Liu J. et al. (2020), who investigated the role of ARID1A in early human cardiac development and neurogenesis. The study demonstrated that homozygous deletion of *ARID1A* in human ESCs results in spontaneous neuronal differentiation due to increased expression of several genes associated with neurodevelopment. Simultaneously, the same cells displayed downregulation of genes associated with cardiomyocyte differentiation (Liu J. et al., 2020).

Han et al. (2019) studied the function of ARID1A in hematopoietic stem cells (HSC), and uncovered that this SWI/SNF subunit is important for the generation of myeloid colonies, for normal T cell maturation, and for the differentiation of both myeloid and lymphoid lineages.

ARID1A loss/gain of function are thought to have context-dependent effects. For instance, *ARID1A* deletion is lethal in early embryonic mouse development (Gao et al., 2008). On the other hand, the depletion of this SWI/SNF subunit induces proliferation of ovarian clear cell carcinoma cells (Xiao et al., 2012; Yamamoto et al., 2012; Lakshminarasimhan et al., 2017). In contrast, another study leveraged a mouse ovarian cancer model and demonstrated that ARID1A loss enhances epithelial differentiation and prolongs survival (Chandler et al., 2015; Zhai et al., 2016). *ARID1A* is instead overexpressed in many hepatocellular carcinomas (Zhao et al., 2016), while the expression of this gene is reduced or lost in colorectal cancer (Mathur et al., 2017).

While there is extensive research investigating the role of ARID1A in cell differentiation, the work performed on ARID1B is thus far limited to a few studies. In this context, Yan et al. (2008) demonstrated that *ARID1B*^–/–^ mouse ESCs are viable but exhibit a slower proliferation rate and tend to spontaneously differentiate. Consistent with this observation, ESCs with homozygous *ARID1B* deletion displayed reduced expression of several pluripotency markers, including *OCT4* and *NANOG*. This suggests that ARID1B may be required to regulate stem cell pluripotency. Recently, Boerstler et al. (2020) leveraged CRISPR/Cas9 to generate a human *ARID1B*-haploinsufficient ESC line with an in-frame deletion of exons 5 and 6 of the gene. Future studies leveraging this cell line may help clarifying the role of ARID1B in pluripotency (Boerstler et al., 2020).

Shi et al. (2020) established *ARID1A* and *ARID1B* deletion mutant lines in zebrafish to investigate the effect of these subunits in neuroblastoma. The authors observed that depletion of ARID1A or ARID1B results in an increased rate of cell proliferation in the sympathoadrenal lineage, which ultimately leads to higher tumor penetrance (Shi et al., 2020).

A zebrafish model was also used to elucidate how ARID1B regulates organismal development (Liu X. et al., 2020). In this study, the authors demonstrated that *ARID1B* haploinsufficiency results in reduced body length due to dysregulated Wnt/β-catenin signaling pathway (Liu X. et al., 2020). An association between ARID1B and the Wnt/β-catenin signaling pathway had already been proposed by Vasileiou et al. (2015).

### ARID1A and ARID1B in Neurodevelopment

ARID1A was associated with neural crest differentiation and craniofacial development (Chandler and Magnuson, 2016). Neural crest cells are a transient, ectoderm-derived, cell population that can migrate throughout the embryo to give origin to craniofacial bone and cartilage, peripheral neurons and glia, melanocytes, and smooth muscle cells (Shakhova and Sommer, 2008). Chandler and Magnuson (2016) generated mice with a conditional, neural crest specific, heterozygous deletion of *ARID1A*. The *ARID1A*-depleted mice displayed craniofacial defects, including shortened snouts and low ears. Additionally, most of the bones involved in the ventral cranial skeleton were greatly reduced in size, leading to abnormal facial features (Chandler and Magnuson, 2016). The study also revealed that conditional haploinsufficiency of *ARID1A* results in defects in developing cardiac neural crest due to an incomplete colonization of the outflow tract and septation of the arterial trunk, ultimately producing defects in the pharyngeal arch arteries. Consistently, homozygous *ARID1A* mutants did not survive *in utero* (Chandler and Magnuson, 2016).

As mentioned, mutations in *ARID1B* often lead to a wide array of neurodevelopmental disorders, including Autism Spectrum Disorders, Coffin-Siris Syndrome, and other forms of Intellectual Disabilities (Vergano et al., 1993; Halgren et al., 2012; Hoyer et al., 2012; Santen et al., 2012; Vals et al., 2014; Yu et al., 2015; Ben-Salem et al., 2016; Sonmez et al., 2016; Jung et al., 2017; Lee et al., 2017; Shibutani et al., 2017; Yu et al., 2018; Demily et al., 2019; Filatova et al., 2019; Pranckeniene et al., 2019; Sekiguchi et al., 2019; van der Sluijs et al., 2019; Curcio et al., 2020; Fujita et al., 2020; Lian et al., 2020; Pascolini et al., 2020; Smith et al., 2020).

Based on these lines of evidence, several studies investigated the role of *ARID1B* in neurodevelopment (Ka et al., 2016; Celen et al., 2017; Jung et al., 2017; Shibutani et al., 2017; Smith et al., 2020). Ka et al. (2016) demonstrated that ARID1B is required for arborization and dendrite growth in cortical and hippocampal pyramidal neurons. *ARID1B* haploinsufficiency resulted in reduced dendritic innervation as well as diminished attachment of dendrites to the pial surface (Ka et al., 2016). In the same study, Ka et al. (2016) found that *ARID1B* mono-allelic loss impairs the formation and maturation of dendritic spines, generating malformations that morphologically resemble those reported in animal models of multiple neuropsychiatric disorders such as ID, ASD, Rett-Syndrome, Down-Syndrome and Fragile-X-Syndrome (Irwin et al., 2002; McKinney et al., 2005; Jentarra et al., 2010; Moffat et al., 2019).

Recently, Smith et al. (2020) leveraged a mouse model to elucidate the consequences of *ARID1B*-haploinsufficiency on the development and function of parvalbumin (PV) and somatostatin (SST) neurons, two of the most prevalent interneuron subtypes. Briefly, the authors discovered that *ARID1B*-haploinsufficiency in PV neurons leads to social and emotional impairments, which are key features of ASD, while *ARID1B* deficiency in the SST population results in learning and memory dysfunction (Smith et al., 2020).

In a similar study, Jung et al. (2017) demonstrated that *ARID1B*-haploinsufficient mice present a reduced number of cortical GABAergic interneurons and decreased proliferation of interneuron progenitors in the ganglionic eminence. These neurological phenotypes are often recovered in autism and schizophrenia patients (Benes and Berretta, 2001; Pizzarelli and Cherubini, 2011). Additionally, in a third mouse model study, Celen et al. (2017) showed that *ARID1B*-haploinsufficient mice are characterized by hydrocephalus, a condition frequently reported also in Coffin-Siris patients (Schrier Vergano et al., 1993). Brain abnormalities were detected in *ARID1B*-haploinsufficient mice also by Shibutani et al. (2017). The *ARID1B*-haploinsufficient mice also exhibited reduced size of the corpus callosum and dentate gyrus, along with impairment in social behavior, altered vocalization, presence of anxiety-like behavior, and growth deficit (Celen et al., 2017).

### ARID1A and ARID1B in Cell Proliferation and Tissue Regeneration

Recent studies conducted on mouse liver linked ARID1A to tissue regeneration (Sun et al., 2016; Li et al., 2019). Specifically, Li et al. (2019) demonstrated that ARID1A is required for the generation of liver-progenitor-like cells (LPLCs) in different types of periportal injuries. In detail, mice with conditional *ARID1A* knockout in the liver displayed impaired LPLCs formation and reduced regeneration of damaged liver tissue (Li et al., 2019). Moreover, *ARID1A*-knockout livers were characterized by significantly increased accumulation of fatty vacuoles and impaired liver function. Conversely, a prior study also performed in the mouse liver demonstrated that suppression of ARID1A is sufficient to promote liver regeneration (Sun et al., 2016). These two studies suggest a dual role for ARID1A in the hepatic context. Sun et al. (2016) proposed a mechanism focused on CYP-metabolism. On the other hand, Li et al. (2019) suggested the presence of a hepatocyte plasticity network where ARID1A promotes the formation of LPLC during injury, while hampering cell proliferation during the recovery stage.

The role of ARID1A and ARID1B in cell proliferation was also investigated using mouse derived preosteoblasts (Nagl Jr., Patsialou et al., 2005; Flores-Alcantar et al., 2011). Notably, Nagl Jr., Patsialou et al. (2005) showed that deletion of *ARID1A* leads to failure in cell cycle arrest. Conversely, the same study demonstrated that loss of ARID1B has not significant impact on the cell cycle (Nagl et al., 2005).

### Molecular Processes Modulated by ARID1A and ARID1B

The SWI/SNF complex is mainly considered as a transcriptional activator, which antagonizes the Polycomb Repressor Complexes (PRC1 and PRC2) in the modulation of gene expression (Kadoch and Crabtree, 2015; Alfert et al., 2019). Nonetheless, repressing activity for the SWI/SNF has also been reported. For instance, a recent study performed on HepG2 cells (hepatocellular carcinoma line) uncovered that ARID1A-containing BAF activates and represses roughly equal numbers of genes (Raab et al., 2015). The same study also demonstrated that ARID1B-containing BAF is primarily a repressor of enhancer activity (Raab et al., 2015). More specifically, Raab et al. (2015) investigated the localization of ARID1A and ARID1B binding sites in HepG2 cells via chromatin immunoprecipitation followed by sequencing (ChIP-seq). The authors observed binding of ARID1A at most enhancers and promoters, while ARID1B was predominantly located at enhancers. Loss of ARID1A from HepG2 cells resulted in a roughly equal number of activated and repressed genes, whereas loss of ARID1B predominantly resulted in transcriptional activation (Raab et al., 2015).

Consistently, ARID1A and ARID1B have been recently associated with acetylation of histone tails at both enhancers and promoters (Chandler et al., 2013; Lei et al., 2015; Raab et al., 2015; Alver et al., 2017; Kelso et al., 2017; Trizzino et al., 2018). For example, Mathur et al. (2017) observed that human *ARID1A*^–/–^ colorectal cancer cells display dampened acetylation levels at Histone H3 lysine 27 (H3K27ac), which is usually associated with transcriptional activity at enhancers and promoters. Correlation between ARID1A loss and attenuation of enhancer acetylation was also observed in zebrafish models (Shi et al., 2020).

Liu X. et al. (2020) profiled chromatin accessibility in wild-type and ARID1A-deleted human ES cells. With these experiments, the authors discovered that loss of ARID1A generated a loss in accessibility at cardiogenic genes, as well as an increase in accessibility at neurogenic genes (Liu X. et al., 2020). These data are thus consistent with a dual (activator/repressor) role of ARID1A in the transcriptional regulation of ESCs. An additional study on human ES cells also revealed that acute depletion of ARID1A increases nucleosome occupancy, and therefore repression, at a set of H3K4me3- and/or H3K27me3-associated promoters (Lei et al., 2015).

The consequences of ARID1A loss on chromatin accessibility were further investigated by Kelso et al. (2017) in colorectal carcinoma lines. The authors demonstrated that loss of ARID1A and ARID1B correlates with global dampening of chromatin accessibility, along with a significant decrease of histone modifications normally associated with transcriptional activation at enhancers (Kelso et al., 2017).

Recently, Wu et al. (2019) linked ARID1A-containing BAF to Condensin, a protein complex involved in the regulation of genomic organization and chromatin looping. The study demonstrated that ovarian cancer cell lines depleted of ARID1A exhibit decreased binding of Condensing-II at active enhancers. Further, they illustrated that ARID1A-loss leads to improper genome compartmentalization (Wu et al., 2019).

Finally, in a recent study conducted in ovarian cancer cell lines, Trizzino et al. (2018) demonstrated that ARID1A and ARID1B play a role in the regulation of RNA Polymerase II promoter-proximal pausing, a widespread mechanism that controls the timing of expression of developmental genes genome-wide.

## Conclusion

In conclusion, multiple lines of evidence point toward a model in which the ARID1A- and ARID1B-containing configurations of the SWI/SNF complex (i.e., the BAF) play an important role in the regulation of pluripotency, as well as in cell fate determination and development (Table 1). Multiple molecular and genomic functions were ascribed to these two SWI/SNF subunits. However, the mechanisms by which ARID1A and ARID1B regulate pluripotency and cell fate are still not fully understood and are likely context-specific. The discovery of such mechanisms, along with the transcription factors and the molecular pathways involved, may open new roads for the diagnosis and the treatment of developmental disorders and cancer.

**TABLE 1:** Biological processes and phenotypes associated to ARID1A and ARID1B.

***STEM CELL PLURIPOTENCY***		
*ARID1A^–/–^* mouse and human ESCs	Failure to maintain pluripotency, spontaneous differentiation, dysregulated expression of pluripotency genes	Gao et al. (2008); Lei et al. (2015), Liu J. et al. (2020)
*ARID1B^–/–^* mouse ESCs	Failure to maintain pluripotency, dysregulated expression of pluripotency genes	Yan et al. (2008)

***CELL DIFFERENTIATION AND PROLIFERATION***		
*ARID1A^–/–^* mouse and human ESCs	Bias toward neuronal differentiation	Gao et al. (2008), Liu J. et al. (2020)
	Failure to differentiate into cardiomyocytes or adipocytes	Gao et al. (2008); Liu J. et al. (2020)
*ARID1A^–/–^* hematopoietic stem cells	Impaired differentiation into myeloid and lymphoid lineages	Han et al. (2019)
*ARID1B^–/–^* Zebrafish	Reduced body length due to dysregulated Wnt/β-catenin signaling	Liu X. et al. (2020)
*ARID1A^+/–^* Zebrafish	Excessive cell proliferation in the sympathoadrenal lineage	Shi et al. (2020)
*ARID1A^–/–^* Mouse (liver)	Impaired liver regeneration, increased vacuole accumulation, liver dysfunction	Li et al. (2019)
*ARID1A^–/–^* Mouse (liver)	Increased liver regeneration	Sun et al. (2016)
*ARID1A^–/–^* Mouse (preosteoblasts)	Dysregulated cell cycle	Nagl Jr., Patsialou et al. (2005)

***DEVELOPMENTAL PHENOTYPES***		
*ARID1A^+/–^* Mouse (neural crest)	Craniofacial defects, shortened snouts, low ears, defects in developing cardiac neural crest	Chandler and Magnuson (2016)
*ARID1B^+/–^* Mouse	Impaired maturation of dendritic spines, reduced dendritic innervation, lack of arborization and dendrite growth in cortical and hippocampal pyramidal neurons	Ka et al. (2016)
	Social and emotional impairments (parvalbumin neurons),	Smith et al. (2020)
	learning and memory dysfunction (somatostatin neurons)	
	Reduced number of cortical GABAergic interneurons,	Jung et al. (2017)
	decreased proliferation of interneuron progenitors in the ganglionic eminence	
	Hydrocephalus, reduced size of the corpus callosum and dentate gyrus, impairment in social behavior, growth deficit	Celen et al. (2017)
	Hydrocephalus	Shibutani et al. (2017)

## Author Contributions

MT and LP designed and wrote the manuscript. Both authors contributed to the article and approved the submitted version.

## Conflict of Interest

The authors declare that the research was conducted in the absence of any commercial or financial relationships that could be construed as a potential conflict of interest.
